# Bite of a Copperbelly Snake

**Published:** 1886-10-20

**Authors:** A. F. Durham

**Affiliations:** Georgia


### Southern Medical Record.

A MONTHLY JOURNAL OF PRACTICAL MEDICINE.

☞All Communications must be addressed to R. C. Word, M. D., Managing Editor.

"*Quicquid Præcipies Esto Brevis.*"

Vol. XVI. ATLANTA, GA., OCTOBER 20, 1886. No. 10

### ORIGINAL AND SELECTED ARTICLES.

### BITE OF A COPPERBELLY SNAKE.

By Dr. A. F. Durham, of Georgia.

About 10 o'clock p. m. on July 27, 1884, Mr. Joe Garrett came to my office and made the following statement of his case :

While seining in a small lagoon he came to a snag, about the middle of the pond, jutting out of the water ; he began feeling around it, on the bottom, with his foot ; while thus engaged he felt a sudden sting on the outer side of his foot ; on moving the foot again he felt the sting a second time ; he paid no special attention to it at the moment, supposing it to be either the prick of a fish fin or brier scratch ; soon, however, the rapid increase of pain led him to suspect a snake bite, and walking out on the bank and inspecting the foot he found four distinct marks of what proved to be snake fangs. Looking across the pond at this time he saw a large brown snake crawl out on the opposite bank and make its way off through the cane brush. It was a copperbelly. He immediately tied a bandage around the leg, took whisky pretty freely, and hastened to see me ; it was about an hour and a half before he arrived at my office.

#### Symptoms.

On his arrival, he presented the following symptoms, viz, Excruciating pains shooting from the wound to the lumbar region, up the spinal column to the head and thence to the chest, the pains shifting every moment with almost the rapidity of lightning; great and incessant nausea and thirst; pupils contracted to almost a bare point; cool, clammy skin; pulse about 65, full, soft and compressible. He remained, with occasional fluctuations, in about this condition nearly or quite thirty-six hours. He was restless during the (Saturday) night, but when quiet and apparently asleep his respiration was slow, rather heavy, and sometimes deep and sighing. On Sunday morning the pains continued so excruciating that ½ grain of sulphate of morphia was given hypodermically. Late in the afternoon, without any premonition, respiration suddenly ceased! Fortunately, just at that moment my friend Dr. A. M. B—— stepped in, and we at once commenced artificial respiration, which was kept up about forty-five minutes, at the end of which time it was found that the patient could breathe voluntarily, and thence forward he had no further trouble in that way. He rested moderately well during the night. On Monday morning the same pains returned, and continued with less severity through the day. He was kept quiet during the (Monday) night by the hypodermic use of morphia. Now comes (to me) the strangest part of the case: About 8 o'clock Tuesday morning he sat up on the cot, which he had all this time occupied, and gazing around him in surprise asked where he was and how came he there! It was perfectly astonishing to me that all this time there had not been the slightest manifestation of mental aberration or impairment of vision.

He appeared perfectly rational, making intelligent replies to all questions asked him, and conversing with visiting friends. Indeed if there was any appearance of mental derangement it was that his mind was preternaturally lucid; he recognized (apparently) every one with whom he was acquainted; he would look at you in conversation, and at any humorous remark would laugh as any one else; but he avered that from the time he reached town until Tuesday morning, he had no remembrance of what had passed, and was totally blind!

### TREATMENT.

The first thing I did was to force all the blood out of the wound I could by scarification, pressure and suction. I then injected into the wound permanganate potass 2 grains to fl ℥ water, and administered a brisk cathartic of calomel, jalap and compound extract colocynth; occasionally administered carbonate ammonia per orem and with hypodermic syringe; and when the pulse was disposed to fag, gave brandy. During the night, had to administer morphia, as before stated, to allay pain. The morning (Sunday) I put him on 9-grain doses iod. potass, alternating with an infusion of chionathus virginicus, Seneca snake root, and sanguinaria canadensis, to each dose of which I added ½ ʒ tincture of lobelia inflata ; also removed bandage, and bathed the limb in a strong solution of chloride of sodium and lime water. During the first three days, I injected, once a day, a weak solution of permanganate of potassium. For several days succeeding Tuesday there was manifest paroxysms of fever, for which I gave bisulphate quinia. For several weeks I kept him on constitutional treatment.

In about six months he had a spell of syncope, once a week, which was always preceded a few minutes by nausea. It was full twelve months before he got entirely rid of the spells. In fact, he says he feels pains in the limbs once a year about the time he was bitten.

I have thus given a succinct account of a very peculiar case, and would like for some medical philosopher to account for the peculiarities in it. Could the treatment have had anything to do with it ? I think not. Memory and vision had been suspended before I did anything for him ; and, as will be seen, the treatment was not rash or heroic after the first few hours. The suspension of memory, sight, and respiration were certainly singular features in the case.

