# Correction

**Published:** 1886-11-20

**Authors:** 


CORRECTION.—In Dr. Durham's article, in our October number, in last paragraph but one, the sentence "In about six months," should read : ***For six months afterwards***, etc.

